# Ferroptosis in pain: evidence, challenges, and opportunities

**DOI:** 10.3389/fimmu.2025.1673783

**Published:** 2025-09-03

**Authors:** Qianqian Yan, Fang Wang, Mengyuan Liu, Jinyan Mao, Zihao Zhao, Bo Wang

**Affiliations:** ^1^ Department of Nephrology, Institute of Kidney Diseases, West China Hospital of Sichuan University, Chengdu, China; ^2^ Nephrology Research Center, The First Affiliated Hospital of Zhengzhou University, Zhengzhou, China; ^3^ Department of Anesthesiology, Air Force Hospital of Western Theater Command, PLA, Chengdu, China; ^4^ Department of Radiology, The First Affiliated Hospital of Zhengzhou University, Zhengzhou, China

**Keywords:** ferroptosis, pain, lipid peroxidation, iron dyshomeostasis, oxidative stress

## Abstract

Pain, an unpleasant but essential feeling for life, affects more than 20% of the global population and burdens health and the economy. Due to the lack of understanding of the exact fundamental mechanism, the existing therapeutic strategies for pain offer finite efficacy and troublesome side effects. The intricacy of the pathogenesis of pain enormously hinders the exploitation of therapeutic approaches. Ferroptosis is a novel mode of cell death characterized by mitochondrial damage, oxidative stress, massive intracellular iron accumulation, and lipid peroxidation. Multiple studies have demonstrated that ferroptosis is involved in the development of pain. We aim to focus on the underlying mechanisms by which ferroptosis participates in pain and recent findings on targeted therapies for ferroptosis in painful diseases. In this review, first, the pivotal mechanisms of ferroptosis are briefly summarized. Second, how ferroptosis participates in pain response is described. Finally, we summarized some substances that relieve pain by alleviating lipid peroxidation and iron accumulation and modulating ferroptosis. We propose that targeting ferroptosis holds tremendous promise in preventing and treating painful diseases.

## 1 Introduction

Pain is a protective response necessary for species survival and a global health problem affecting human life, health, and social development (1). The International Association for the Study of Pain (IASP) recently defined pain as unpleasant sensory and emotional experiences related to actual or potential tissue damage or resembling such damage (2). Based on duration, pain can be divided into acute pain (which has survival value and is implicated in healing) and chronic pain (often considered a disease); chronic pain alone affects more than 30% of the global population, placing an enormous economic burden on individuals and society (3, 4). Pain varies widely in quality, intensity, duration, pathophysiologic mechanisms, and meanings; however, all are signaled by precise receptors and a system of fibers that extend from the periphery to the brain, alarming tissue injury (2, 5). Drugs and strategies for pain treatment are diverse, but some problems remain that need to be addressed in the field, such as the limited number of existing analgesics, poor analgesic efficacy, adverse reactions and social harms of therapeutics, and lack of original innovation in pain research mechanisms (6, 7). Optimizing the therapeutic effect of drugs and exploring the new mechanism of pain to find a new analgesic program has become an expectant problem in the clinical treatment of pain.

Ferroptosis, a unique form of oxidation and iron-driven programmed cell death, has been demonstrated to participate in various types of pain (8–10). Because it usually does not exhibit alterations in nuclear morphology or caspase-3 activity and is primarily uninhibited by caspase suppressors, ferroptosis is easily distinguished from other types of cell death, such as necrotic necroptosis, apoptosis, and autophagy (11, 12). Mitochondrial contraction, membrane concentration, and crista reduction or vanishment are generally considered to be ultrastructural signs of ferroptosis (13). Initially, Dixon et al. discovered that ferroptosis mainly involves three metabolic substances, including thiols, lipids, and iron, which produce iron-dependent lipid peroxides and ultimately cause cell death (12, 14). When ferroptosis occurs, transferrin increases iron intake through transferrin receptors; subsequently, excess iron generates reactive oxygen species (ROS) and activates iron-containing enzymes, causing lipid peroxidation, ultimately resulting in oxidative membrane damage (5). Some bioactive molecules regulate ferroptosis by directly or indirectly influencing iron metabolism and lipid peroxidation, such as glutathione peroxidase 4 (GPX4), nuclear factor E2 associated factor 2 (NRF2), heat shock protein beta-1(HSPB1), NADPH oxidase (NOX), p53, etc. (8, 13, 15). Studies have highlighted that ferroptosis is involved in the occurrence and development of numerous diseases, including neurological diseases, systemic inflammatory autoimmune diseases, cardiovascular diseases, and so on (16–18). In addition, recent studies have found ferroptosis is also associated with pain, as Guo et al. injected ferroptosis inhibitors into the rats’ peritoneum, attenuating their chronic sciatic nerve pain (19). In bone cancer pain (BCP) mice, the ferroptosis inhibitor (ferrostatin-1, Fer-1) hindered iron accumulation, reduced GPX4 activity, and alleviated BCP-induced lipid peroxidation (8).

Over the past few years, numerous studies on the correlation between pain and ferroptosis have exploded, making the mechanisms involved increasingly clear. This review will present the latest research progress between ferroptosis and pain in recent years, exploring pivotal findings and open questions to design more efficacious therapeutic approaches for pain.

## 2 Mechanisms of ferroptosis

### 2.1 Lipid peroxidation

**Figure 1:**
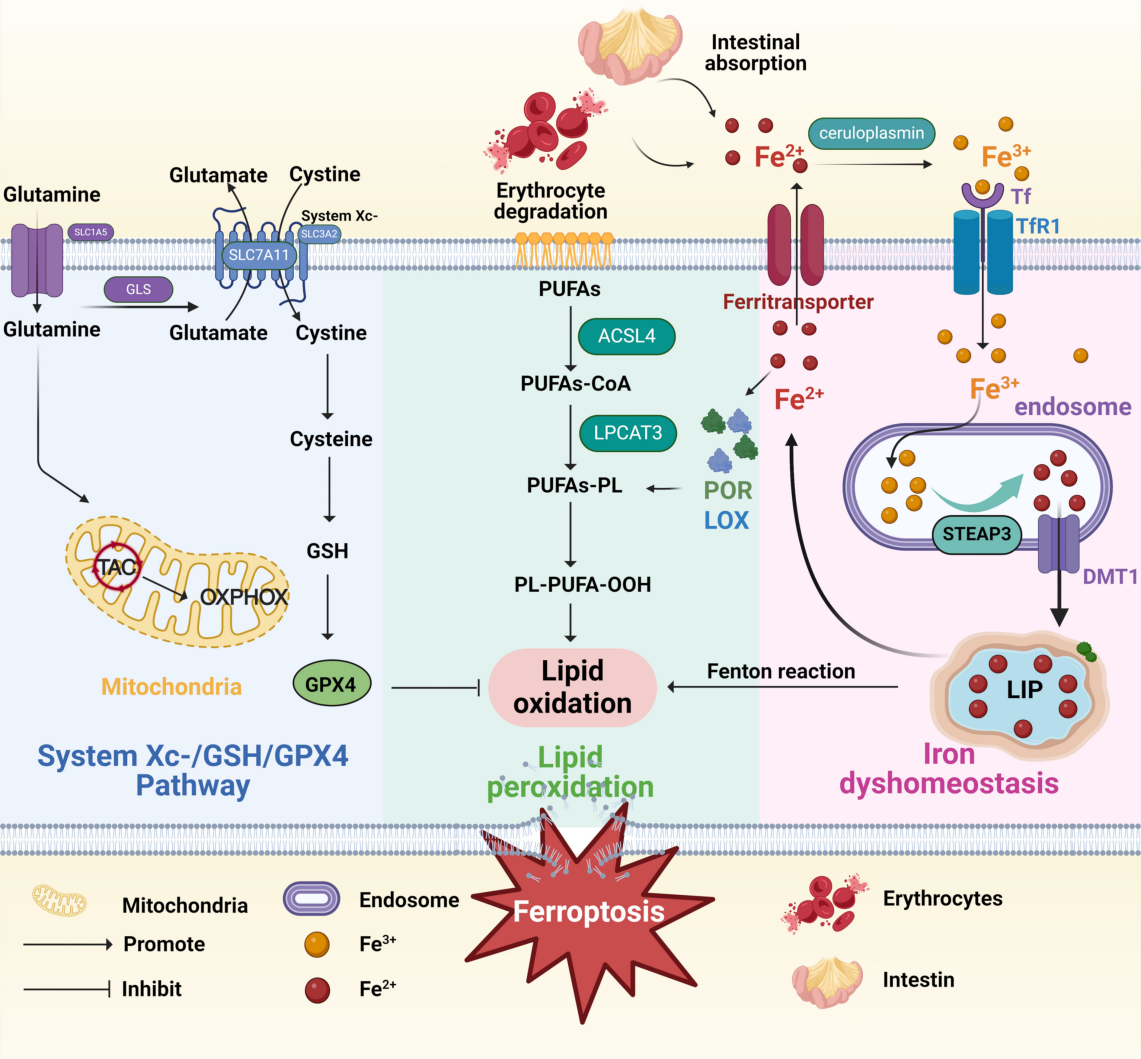
Mechanisms of ferroptosis. The figure shows mechanisms of ferroptosis induced by three pathways, including System Xc-/GSH/GPX4 Pathway (the blue box), lipid peroxidation (the green box), and iron dyshomeostasis (the pink box). The System Xc-/GSH/GPX4 Pathway: Cystine enters the cell through the system Xc- and is then converted into cysteine to promote GSH synthesis and ultimately inhibit ferroptosis. The lipid peroxidation: ACSL4 catalyzes PUFAs to bind CoA to form PUFA-CoA, which is further inserted into membrane PLs under the action of LPCAT3 to form PUFA-PL. Large amounts of PUFAs oxidize and lipify to produce PL-PUFA-OOH and ROS, predisposing the cells to ferroptosis. The iron dyshomeostasis: Ceruloplasmin oxidizes Fe^2+^ to Fe^3+^, which binds to Tf and endocytoses into cells through the action of TfR1. After the release from Tf, Fe^3+^ is reduced to Fe^2+^ through STEAP3 and transferred from the endosome/lysosome to the cytosol via apical DMT1. The iron present in the cytoplasm can be stored by ferritin as iron ions or in the transient LIP to eliminate cytotoxicity; on the other hand, it can be transported outside the cell by ferritransporters. TAC, tricarboxylic acid cycle; GLS, glutaminase; OXPHOX, oxidative phosphorylation; GSH, glutathione; GPX4, glutathione peroxidase 4; PUFAs, polyunsaturated fatty acids; ACSL4, Acyl-CoA synthase long-chain family member 4; PLs, phospholipids; LPCAT3, lysophosphatidylcholine acyltransferase 3; PL-PUFA-OOH, phospholipid hydroperoxides; Tf, transferrin; TfR1, transferrin receptor 1; STEAP3, six-transmembrane epithelial antigen of prostatic 3; DMT1, divalent metal transporter 1; LIP, labile iron pool. Created in BioRender.

Lipid peroxidation of polyunsaturated fatty acids (PUFAs) in cell membranes is a crucial feature of ferroptosis (20). PUFAs are the major targets of peroxidation, especially arachidonic acid and epinephrine (20, 21). Lipid peroxidation involves a series of multifaceted events, including initiating reactive oxygen species, propagating chain and chain-branched reactions, and terminating free radical reactions (16). As essential substrates of lipid metabolism, PUFAs experience esterification into membrane phospholipids and oxidation, ultimately promoting the transmission of ferroptosis signals (**Figure 1**) (22–24). In addition, Acyl-CoA synthase long-chain family member 4 (ACSL4) catalyzes PUFAs to bind CoA to form PUFA-CoA, which is further inserted into membrane phospholipids (PLs) under the action of lysophosphatidylcholine acyltransferase 3 (LPCAT3) to form PUFA-PL (25, 26). Large amounts of PUFAs oxidize and lipify to produce phospholipid hydroperoxides (PL-PUFA-OOH) and ROS, predisposing the cells to ferroptosis (16, 27). Continuous lipid peroxidation causes a breakdown of membrane integrity, eventually leading to the plasma membrane fracture (27).

### 2.2 Iron dyshomeostasis

Iron is a significant material basis for metabolic processes, including mitochondrial respiration, DNA synthesis, and cell signaling, but ferroptosis may occur when it accumulates excessively (28). Iron is present in the body’s local microenvironment in two forms: ferrous (Fe^2+^) and ferric (Fe^3+^) ions (**Figure 1**) (29). Fe^2+^, primarily generated from erythrocyte degradation and intestinal absorption, can enhance ROS production through the Fenton reaction, thus facilitating lipid peroxidation (30). *In vivo*, free Fe^2+^ enhances the activity of lipoxygenase (LOX) and cytochrome P450 oxidoreductase (POR), enzymes that catalyze polyunsaturated fatty acid-containing phospholipids (PUFA-PLs) oxidation, thereby affecting lipid peroxidation (14). In addition, ceruloplasmin oxidizes Fe^2+^ to Fe^3+^, which binds to transferrin (Tf) and endocytosis into cells through the action of transferrin receptor 1 (TfR1) (31). After the release from Tf, Fe^3+^ is reduced to Fe^2+^ through six-transmembrane epithelial antigen of prostatic 3 (STEAP3) and transferred from endosome/lysosome to the cytosol via apical bivalent metal transporter 1 (DMT1) (32). The iron present in the cytoplasm can be transported to the corresponding site to play its role. On the one hand, it can be stored by ferritin as iron ions or in the transient labile iron pool (LIP) to eliminate cytotoxicity; on the other hand, it can be transported outside the cell by ferritransporters (33). Overall, free intracellular iron exceeding a certain level triggers ferroptosis. Excess iron in cells increases the level of Fe^2+^, thus promoting lipid peroxidation through the Fenton reaction, and the heightened ROS eventually induces ferroptosis (34).

### 2.3 System Xc-/GSH/GPX4 pathway

Glutathione (GSH), which mainly includes cysteine, glutamic acid, and glycine, is a vital inhibitor and endogenous antioxidant in ferroptosis (35). In the body, an appropriate amount of GSH can effectively offset the increased ROS, thus acting as an elementary defense mechanism to protect cells from various forms of oxidative stress (OS) (36). Once the GSH-dependent lipid peroxide repair systems are damaged, ROS accumulate within the body, promoting ferroptosis (16, 27). Cystine-glutamate antiporter (System Xc−), a disulfide-linked heterodimer consisting of two subunits, SLC3A2 (the regulatory subunit 4F2hc) and SLC7A11 (the transporter subunit xCT) (**Figure 1**) (37, 38). xCT transits one cystine into the cell and one glutamate out of the cell, while 4F2hc stabilizes the xCT and assists in its subcellular localization (39, 40). Once cystine enters the cytoplasm, it is rapidly transformed into cysteine, an essential step for GSH production (16). Therefore, xCT regulates the production of GSH and, together with GSH, protects cells from oxidative damage. GPX4, the only member of the selenium-dependent glutathione peroxidase family, uses GSH as cofactors to convert toxic lipid peroxides on phospholipid membranes to nontoxic lipid alcohols (41, 42). GSH depletion can directly or indirectly result in the decrease of GPX4 activity and then induce ferroptosis (43). Unlike GSH, RSL3 (the first discovered ferroptotic compounds) and wathaferin A directly mediate GPX4’s inactivation or depletion (44).

## 3 Correlation between ferroptosis and pain

**Figure 2:**
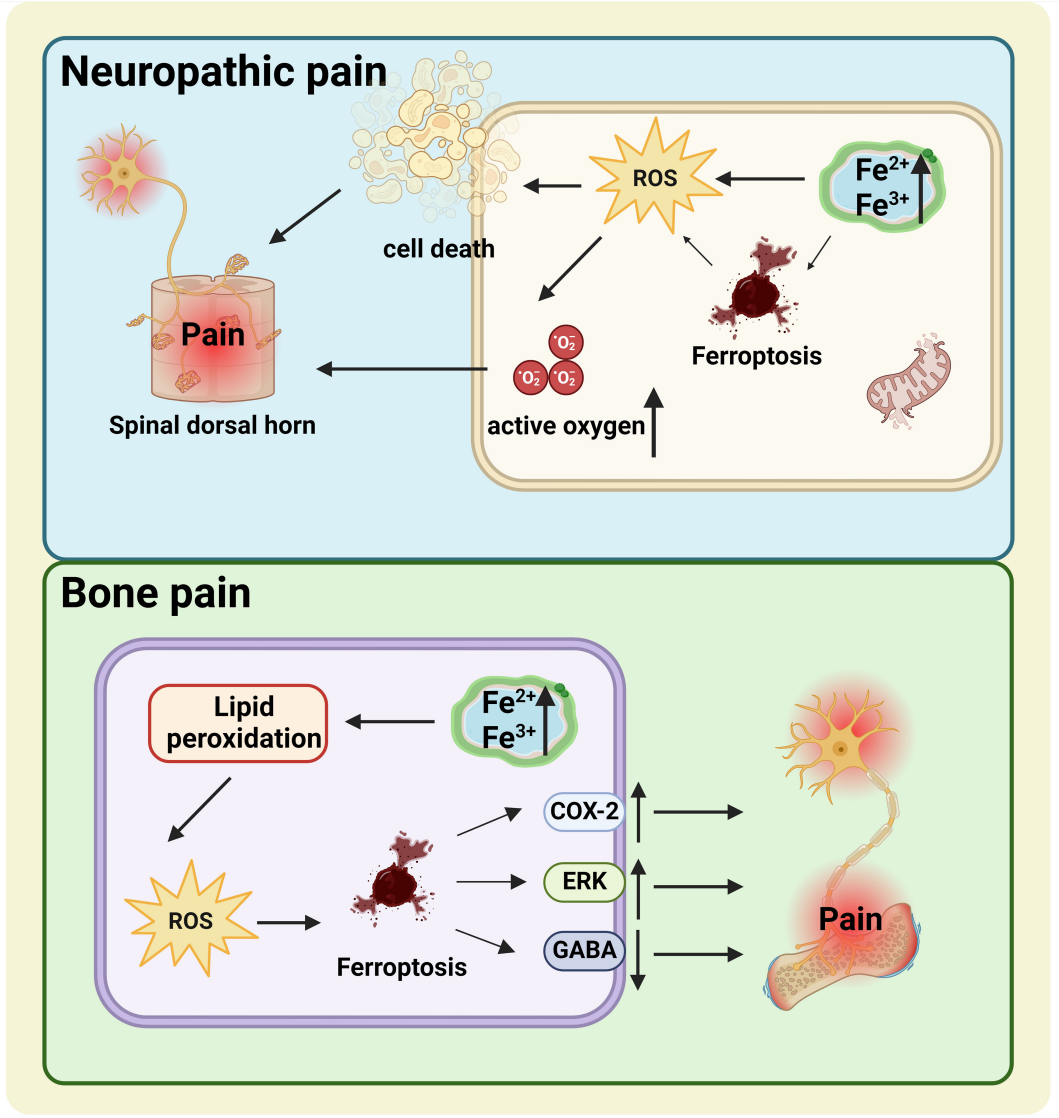
The relationship between ferroptosis and NP (the blue box) and BP (the green box). NP, Elevated iron ions in neurons of the damaged nervous system increase the risk of ferroptosis. Increased ROS levels during ferroptosis destroy the integrity of the cell membrane and cause NP. BP, The upregulation of COX-2 and ERK and the downregulation of GABA caused by cell ferroptosis may be important mechanisms of BP. NP, neuropathic pain; BP, bone pain; ROS, reactive oxygen species.

Previous studies have demonstrated the relationship between ferroptosis and neuropathic pain (NP) and bone pain (BP) (**Figure 2**) (8–10, 19). Unfortunately, the exact mechanism by which ferroptosis functions in these pains has not yet been fully established. NP, a type of chronic pain, is directly caused by trauma or disease involving the somatosensory system (45). A persuasive NP-causing factor is neuronal apoptosis or damage mediated by peripheral nerve or spinal cord injury (46). Some researchers believe that elevated iron ions in neurons of the damaged nervous system increase the risk of ferroptosis (46). Increased ROS levels during ferroptosis destroy the integrity of the cell membrane (47). Does the ROS produced by ferroptosis damage the cell membrane of nerve cells? It has been shown that elevated ROS-related oxidation activity is correlated with NP and plays a vital role in NP processes (48). Intracellular ROS activation may generate cell death, including in spinal dorsal horn neurons, presumably a crucial reason for ROS involvement in NP (45, 49). In the rat model of chronic contractile injury (CCI), ferroptosis was found to block neuron and astrocyte activation in the dorsal horn of the spinal cord to participate in the occurrence of NP (46). Similarly, Ding et al. found hallmarks of ferroptosis in the spinal dorsal horn of BCP mice, including mitochondrial morphological changes, iron accumulation, and lipid peroxidation (8). Therefore, ferroptosis is thought to participate in the development and maintenance of NP by blocking the activity of spinal dorsal horn neurons and astrocytes (46). These results all prove that ferroptosis is associated with the development of pain.

Analogously, increased iron levels were detected in the spinal cords of BCP mice (8). BCP, manifested as spontaneous persistent pain, may develop due to the destruction of dorsal horn neurons in the spinal cord (50, 51). One possible reason is decreased production of the inhibitory neurotransmitter gamma-aminobutyric acid (GABA) (52). In other words, when GABAergic neurons are lost in the dorsal horn of the spinal cord, pain behavior increases. GABAergic inhibitory interneurons have high energy demands and are especially vulnerable to OS, hypoxia, and glutamate accumulation (53, 54). Multiple studies have suggested that OS may be responsible for reducing GABaergic neurons in kinds of neurological diseases (8). Coincidentally, ferroptosis is driven by the excessive ROS caused by lipid peroxidation, which is closely related to OS (55). Lipid peroxidation and OS conduce to continuous chronic pain (8, 46, 56). The buildup of lipid ROS is primarily attributed to the disappearance of the GPX4 effect (57). GPX4 levels can be directly or indirectly affected by other compounds involving a variety of pathways, of which the extracellular regulated protein kinases (ERK) pathway is closely associated with neural cell ferroptosis (8, 58). Cyclooxygenase-2 (COX-2), a biomarker for ferroptosis, is a crucial derivable polypeptide mediating pain and inflammation (59, 60). Research has confirmed that COX-2 and p-ERK1/2 levels were significantly increased in BCP mice (8). Thus, the idea that ferroptosis is associated with pain-related COX-2 activation and ERK pathways in BCP is scientific and rigorous. Ferroptosis is a feasible therapeutic target for patients with chronic pain. A deeper exploration of the fundamental mechanisms involved is crucial to developing effective pain treatments.

## 4 Ferroptosis and pain-related illness

### 4.1 Neuropathic pain-related diseases

#### 4.1.1 Multiple sclerosis

**Figure 3:**
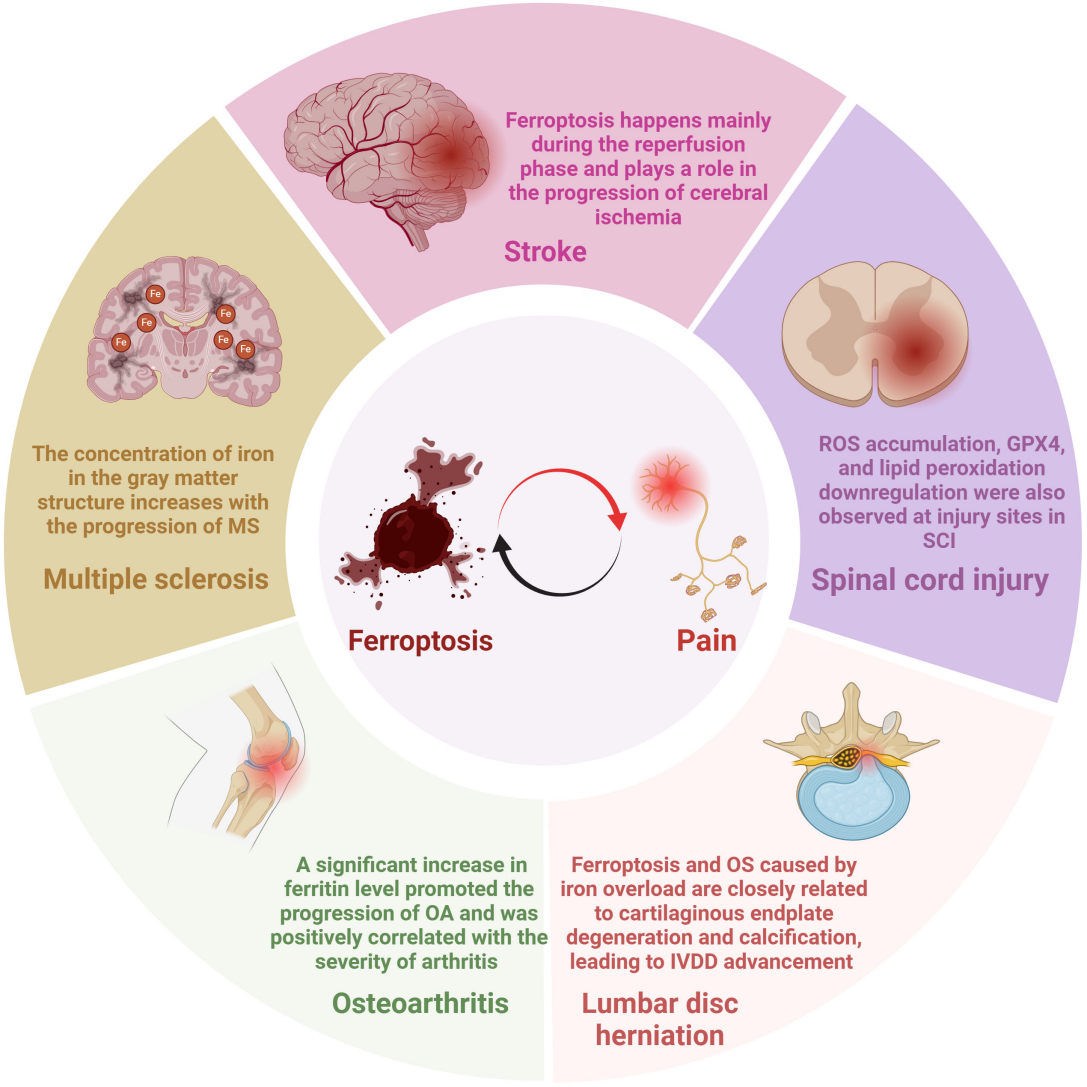
Mechanisms of ferroptosis in pain-related diseases.

**Figure 4:**
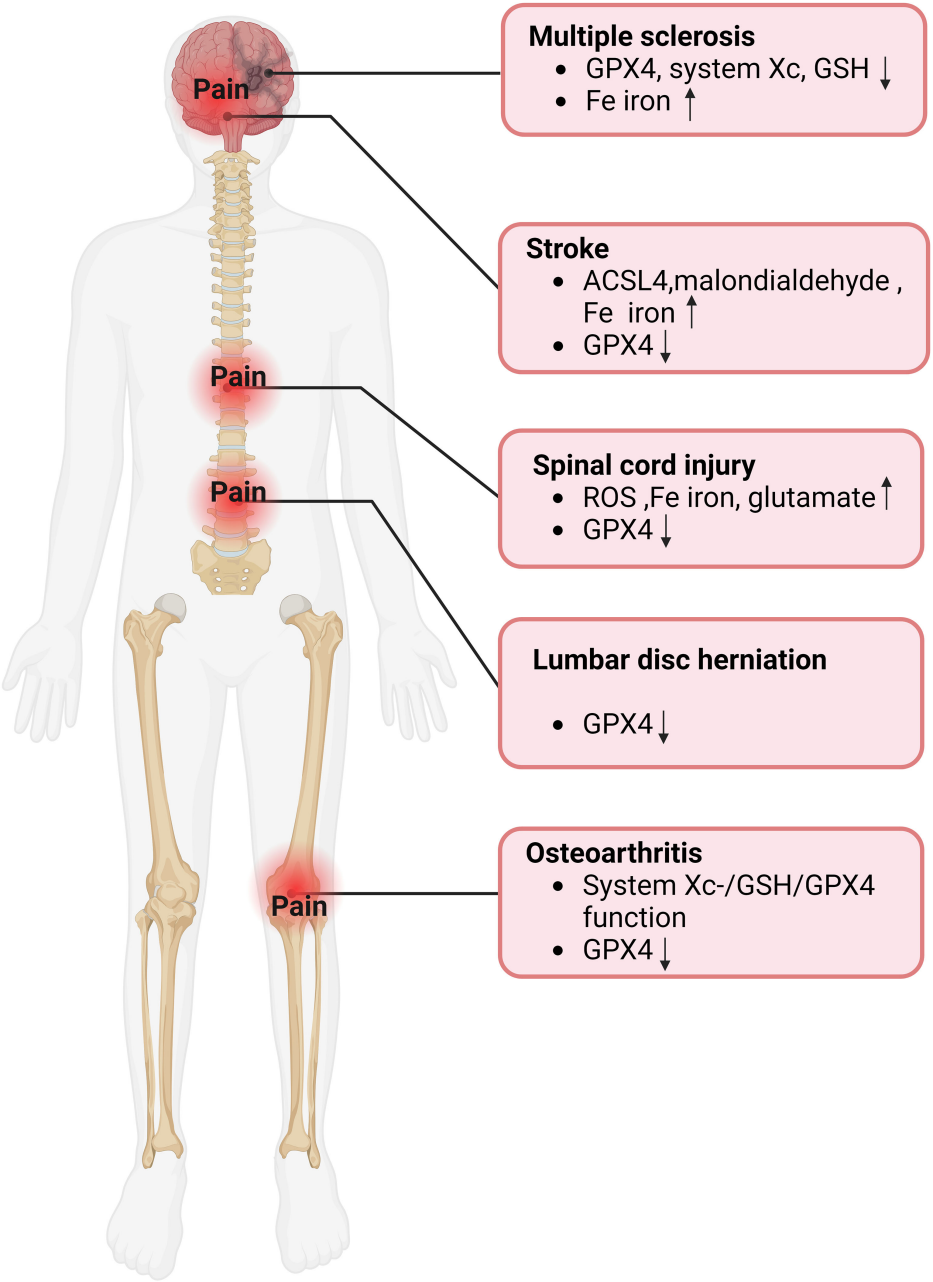
Evidence of ferroptosis in pain-related diseases. GPX4, glutathione peroxidase 4; GSH, Glutathione; ACSL4, Acyl-CoA synthase long-chain family member 4; ROS, reactive oxygen species.

Multiple sclerosis (MS), a chronic inflammatory and demyelinating disease with the most common and severe symptoms of NP and spasticity, affects approximately 2.1 million people worldwide (61–63). As early as fifteen years ago, brain magnetic resonance imaging (MRI) scans displayed that the concentration of iron in the gray matter structure increases with the progression of MS (**Figure 3**) (64). In recent years, the idea of ferroptosis participants in the MS process has been repeatedly mentioned and increasingly accepted (65, 66). Hu et al. observed in an MS animal model that mRNA levels of all three GPX4 isoforms (cytoplasmic, mitochondrial, and nuclear) decreased in the MS gray matter (**Figure 4**) (65). To further support the hypothesis, the research found that other biomarkers involved in ferroptosis, such as system Xc and GSH, were also significantly reduced in this animal model (63, 65). In addition, a clinical trial study on MS has shown that iron chelators can delay the disease progression (67). At different stages of MS, ferroptosis affects its development through diverse mechanisms. In the early stages of MS, ferroptosis interacts with multiple cell types, including macrophages and microglia, resulting in cellular oxidative damage and cytotoxicity (68). Microglia, as the primary iron-handling cells in the central nervous system, demonstrate exceptional capacity for iron accumulation and storage. Iron overload can cause inflammatory phenotype of microglia, resulting in the production of proinflammatory cytokines and contributing to neurodegeneration (69). With the progression of MS, the dysregulation of the SystemXc-GSH-GPX4 pathway, which affects oligodendrocyte death and demyelination, has become a more critical factor impacting MS (70). In summary, ferroptosis functions in MS regulation and has excellent potential as a therapeutic target.

#### 4.1.2 Stroke

The number of new strokes each year is enormous, with about 12 million in 2019, and about 11% of stroke patients develop central post-stroke pain (CPSP) (71, 72). CPSP, a disabling and intractable NP syndrome, is the most frequent form of central NP all over the world (73–75). Ischemic stroke (IS), which accounts for more than 80% of all stroke events, is modulated by ferroptosis (76, 77). Cerebral blood flow obstruction is the primary pathological mechanism of IS, while brain damage persists and continues to progress during reperfusion (78, 79). Interestingly, studies have indicated that ferroptosis happens mainly during the reperfusion phase and plays a role in the progression of cerebral ischemia (**Figure 3**) (80). Specifically, with the extension of reperfusion time, ACSL4 level, iron content, and malondialdehyde level gradually increased, while GPX4 level decreased, but all the above indexes did not change significantly during ischemia (**Figure 4**) (81). Ischemia reduces the supply of oxygen and nutrients to brain tissue, triggering OS and inflammation, directly or indirectly influencing metal ion metabolism, including iron ions (82, 83). Due to the destruction of the blood-brain barrier and increased vascular permeability, iron content in the brain tissue of stroke patients is significantly increased, especially around the infarction (83, 84). In addition, serum iron levels are associated with an increased risk of IS and a lousy outcome (79, 85). Although ferroptosis is involved in secondary brain injury associated with IS, the current understanding of its role in IS is still limited, and its underlying pathological mechanisms remain to be elucidated.

#### 4.1.3 Spinal cord injury

Spinal cord injuries (SCI) often contribute to widespread sensorimotor and autonomic nerve damage, affecting about 2.5 million people worldwide (86, 87). Chronic pain is an ordinary complication after SCI, accounting for about 61% of all patients, while NP is considered the most severe pain post-SCI, with an estimated prevalence of 53% reported (88, 89). The molecular mechanism of SCI remains not entirely explicit, so there is no effective therapy for SCI in the clinic (87). It has been verified that ferroptosis is involved in the pathophysiological process of SCI, mainly manifested as ROS increased, iron overload, glutamate, and lipid peroxidation accumulation related to ferroptosis in SCI (**Figure 4**) (90). The concentration of iron in the spinal cord tissue of SCI rats was significantly increased for more than a week, and mitochondrial morphological characteristics of ferroptosis were observed (91). In addition, ROS accumulation, GPX4, and lipid peroxidation downregulation were also observed at injury sites in SCI mouse models (**Figure 3**) (92). Evidence of the involvement of ferroptosis in SCI appeared not only in the spinal cord of animal models but also in the significant increase in iron deposition and iron-induced lipid ROS accumulation detected in the intracranial motor cortex of SCI rats (93). Neurons and oligodendrocytes are the main types of nerve cells involved in ferroptosis (94, 95). Ferroptosis of neurons leads to structural atrophy of neurons in the motor cortex accompanied by the death of corresponding axons (93, 94). This ultimately shortens the number of neurons in the motor cortex, a crucial reason for the problematic restoration of function in patients with SCI (93, 94). Notably, pain’s activity-dependent sensitization occurs in peripheral receptors, neurons, and glial cells (96). The overlap of action sites further proves a strong link between pain and ferroptosis. Similar to the previous two diseases, extensive literature has proved that ferroptosis is closely related to SCI. However, there is no sufficient specific explanation of its involvement in the pathological process of SCI and the possible problems in the clinical conversion of ferroptosis inhibitors.

### 4.2 Bone pain-related diseases

#### 4.2.1 Osteoarthritis

Osteoarthritis (OA), an intricate and heterogeneous disease affecting multiple joints, causes chronic pain and permanent damage to the body’s joints (97, 98). The prevalence of OA continues to rise, affecting an estimated 303 million adults worldwide (99). Despite the high prevalence of OA, the mechanisms of OA-related pain are still not fully understood, so prescription drugs suggested by international guidelines for OA management can only relieve the pain for symptoms (99–101). A significant increase in ferritin level promoted the progression of OA and was positively correlated with the severity of arthritis (**Figure 3**) (102, 103). The joint synovium and subchondral bone are the main sites of origin for OA-related pain (99). When these areas are subjected to harmful mechanical, chemical, and other stimuli, these harmful inputs are converted into electrical signals that travel along the spinal cord’s dorsal root ganglion and dorsal horn to the brain, causing pain (99). Zhang et al. demonstrated the critical role of chondrocyte ferroptosis in the development of OA for the first time (104). Ferroptosis in chondrocytes increased the expression of matrix-degrading enzymes but decreased collagen II expression (99). Collagen II constitutes the extracellular matrix (ECM), which, together with chondrocytes, is the main component of articular cartilage (105). The System Xc-/GSH/GPX4 pathway is critical in the pathogenesis of chondrocyte ferroptosis. Researchers discovered ferroptosis-related morphological alterations, including mitochondrial contraction and mitochondrial membrane thickening in cartilage samples from OA patients (106). In addition, GPX4 protein expression was markedly decreased in the cartilage tissue of the OA mouse model (**Figure 4**) (106). In the progression of OA, GPX4, on the one hand, regulates ferroptosis or OS in chondrocytes and, on the other hand, promotes ECM degradation via the MAPK/NF-KB signaling pathway (107).

#### 4.2.2 Lumbar disc herniation

A common reason for chronic low back pain is lumbar disc herniation, which influences 70-85% of the global population (108). One of the leading causes of lumbar disc herniation is intervertebral disc degeneration (IVDD), which can be triggered by the OS of nucleus pulposus cells and degeneration of cartilage endplates (109, 110). Interestingly, recent research proposes that ferroptosis may also be innegligible in this process (111). The specific manifestations were down-regulated GPX4 expression in disc tissue of patients with disc degeneration (**Figure 4**) (112). In addition, morphological features of ferroptosis, such as dense and shrunken mitochondria, were observed in *in vitro* cell models of IVDD, and ferroptosis inhibitors could reverse these effects (112). Like articular cartilage, chondrocytes are also the principal cells of the cartilage endplate, which transport nutrients to the intervertebral disc (113). Studies have indicated that ferroptosis and OS caused by iron overload are closely related to cartilaginous endplate degeneration and calcification, leading to IVDD advancement (**Figure 3**) (114, 115). It is worth noting that although most studies have not observed the presence of iron deposition in the nucleus pulposus, since iron deposition in the human body is a long process, focusing on iron’s role in the nucleus pulposus may benefit patients (113). The studies mentioned above have validated the link between disc degeneration and ferroptosis, and more research should focus on the specific mechanisms involved.

In addition, emerging evidence implicates ferroptosis functions in rheumatoid arthritis (RA) and osteosarcoma pathogenesis, both of which can manifest clinically as significant bone pain (116, 117). Ferroptosis contributes to articular cartilage destruction in RA, and Yang et al. developed a hydrogel-based therapeutic strategy that protects chondrocytes from ferroptosis (117). What is particularly noteworthy is that ferroptosis compromises the survival ability of osteosarcoma cells, and inducing ferroptosis significantly increases their sensitivity to cisplatin (116, 118).

Much of the specific mechanism that ferroptosis participates in pain-related diseases has not been fully elucidated. Exploring the mechanisms involved could be a promising way to develop new methods for ferroptosis in painful diseases.

## 5 New methods for pain-related illness treatment based on ferroptosis

As the link between ferroptosis and pain is widely revealed, researchers are pyramidally concentrating on seeking novel means of pain treatment based on ferroptosis, aiming to explore more potential targets for pain easement (**Table 1**).

**Table 1:** Summary of the therapeutic agents targeting ferroptosis to treat pain.

Pain	Agents/methods	Model	Administration	Mechanism	Target	Conclusion	References
NP	VitD3	SNI‐induced rat model	Intrathecal	Inhibits PKCa/NOX4 signaling pathway	Oxidative metabolism	VitD presents an innovative therapeutic avenue for NP.	(120)
	Ad-Sirt2	SNI‐induced rat model	Intrathecal	Upregulates the expression of Sirt2	Lipid peroxidation	Sirt2-targeted therapeutics may help relieve the symptoms of chronic NP.	(10)
	Liproxstatin-1	CCI-induced rat model	Intrathecal	Restores the dysregulations in GPX4 and ACSL4 levels	Lipid peroxidation	Liproxstatin-1 has the potential to inhibit ferroptosis-like cell death and attenuate NP.	(19)
	Fingolimod	Mitochondrial oxidative damage neuronal model	Cell culture	Increases mitochondrial stability and restores mitochondrial dynamics	Oxidative stress	Fingolimod is a potential candidate for the treatment of neurodegenerative diseases.	(129)
	DMF	Chronic cerebral hypoperfusion-induced rat model	Intragastric administration	Regulates ferroptosis through the Nrf2/ARE/NF-kB signaling pathway	Oxidative stress	DMF has a neuroprotective function.	(130)
	Prokineticin-2	CCI-induced mice model	Intracerebroventricular injection	Regulates iron and lipid peroxidation	Lipid peroxidation	Prokineticin-2 mediates neuronal cell deaths in traumatic brain injury via ferroptosis.	(131)
	Ferrostatin-1	Focal cerebral ischemia mice model	Intranasal	Activates the AKT/GSK3β pathway	Iron content	Ferroptosis may become a novel target in the treatment of IS.	(133)
	Selenium	Mouse middle cerebral artery occlusion model	Intraperitoneal injection	Upregulates Mfn1 expression	Oxidative stress	Selenium alleviates OS and ferroptosis by promoting mitochondrial fusion.	(134)
	Edaravone	Rats middle cerebral artery occlusion and reperfusion model	Intraperitoneal administration	Activates the Nrf2/FPN pathway	Oxidative stress	Edaravone inhibits ferroptosis to attenuate cerebral ischemic reperfusion injury.	(135)
	Galangin	Hippocampal neurons underwent oxygen-glucose deprivation	Cell culture	Activates the SLC7A11/GPX4 axis	Oxidative stress	Galangin inhibits ferroptosis of hippocampal neurons.	(136)
	SM	Transient middle cerebral artery occlusion mice model	Oral administration	Regulates lipid peroxidation	Lipid peroxidation	SM provides neuroprotection against IS and has the potential as a therapeutic agent.	(137)
	EA	CCI-induced rat model	EA treatment	Regulates the SAT1/ALOX15 pathway	Lipid peroxidation	EA inhibited ferroptosis by regulating the SAT1/ALOX15 pathway to treat NP.	(139)
BP	D-mannose	Anterior cruciate ligament transection‐induced OA mice model	Oral administration	Upregulates HIF‐2α expression	Immunomodulation	D‐mannose alleviates OA progression by suppressing HIF‐2α‐mediated chondrocyte sensitivity to ferroptosis.	(148)
	Quercetin	Anterior cruciate ligament transection‐induced rat model	Intra-articular injections	Activates the Sirt1/Nrf−2/HO−1	Oxidative damage	Quercetin can effectively alleviate OA progression in rats.	(149)
	Dandouchi (Vitamin K2)	Anterior cruciate ligament transection‐induced rat mouse model	Intra-articular injections	Inhibits the MAPK/NFκB signaling pathway	Lipid peroxidation	Vitamin K2 inhibits the MAPK/NFκB signaling pathway to decelerate the degradation of the chondrocyte extracellular matrix.	(150)
	JTF	Enzyme papain-induced rats model	Intragastric route	Inhibits NCOA4-HMGB1-	Inflammatory cytokines	JTF exerts cartilage protective and anti-edema effects in OA therapy.	(151)
	LXA_4_	Monosodium iodoacetate-induced OA	intraperitoneal	Activates the ESR2/LPAR3/Nrf2 axis	Inflammatory responses	LXA_4_ inhibited ferroptosis, thereby alleviating the pain and pathological progression of OA.	(152)
	BONT/A	Monosodium iodoacetate-induced rats model	injection by intra-articular route	Regulates SLC7Al1/GPX4 axis	Oxidative damage	BONT/A alleviates the degradation of cartilage and OA development by inhibiting ferroptosis of chondrocytes.	(153)
	MNP	Medial meniscus-induced mouse model	intra-articular injection	Targets TRPV1 pathogenesis	Inflammatory responses	An intelligent TRPV1 targeting bifunctional magnetothermal switch for the pathogenesis-based precise OA therapy.	(154)
	colloidal gold nanorods	Medial meniscus-induced mouse model	intra-articular injection	Activates of TRPV1 signaling	Attenuate chondrocyte ferroptosis	Citrate‐stabilized gold nanorods improved physical activities and alleviated the pain of destabilization of the medial meniscus OA mice.	(155)
	reactive oxygen species-responsive hydrogel	Rat-tail vertebra IVDD model	Intradiscal injections	Upregulates SLC7A11 expression	Oxidative damage	An injectable reactive oxygen species-responsive hydrogel represents a potential novel treatment strategy for patients with early-stage IVDD.	(156)
	glutamine	IDD Sprague–Dawley rats model	Intradiscal injections	Deubiquitination of Nrf2	Lipid peroxidation	Glutamine is a promising novel therapeutic target for the management of intervertebral disc degeneration.	(157)
	Tinoridine	IVDD Sprague–Dawley rats model	Intradiscal injections	Regulates the Nrf2/GPX4 pathway	Oxidative damage	Tinoridine is a novel ferroptosis inhibitor for potential intervertebral disc degeneration therapy.	(158)

NP, neuropathic pain; BP, bone pain; VitD3, Vitamin D3; SNI, spared nerve injury; Ad-Sirt2, adenoviruses overexpressing sirtuin 2; CCI, chronic constriction injury; DMF, dimethyl fumarate; SM, salvia miltiorrhiza; JTF, Jianpi-Tongluo Formula; LXA4, lipoxin A_4_; BONT/A, botulinum toxin A; MNP, magnetic nanoparticles; IVDD, intervertebral disc degeneration; IDD, intervertebral disc degeneration; IS, ischemic stroke; OS, oxidative stress; EA, electroacupuncture; OA, osteoarthritis.

### 5.1 Neuropathic pain-related diseases

**Figure 5:**
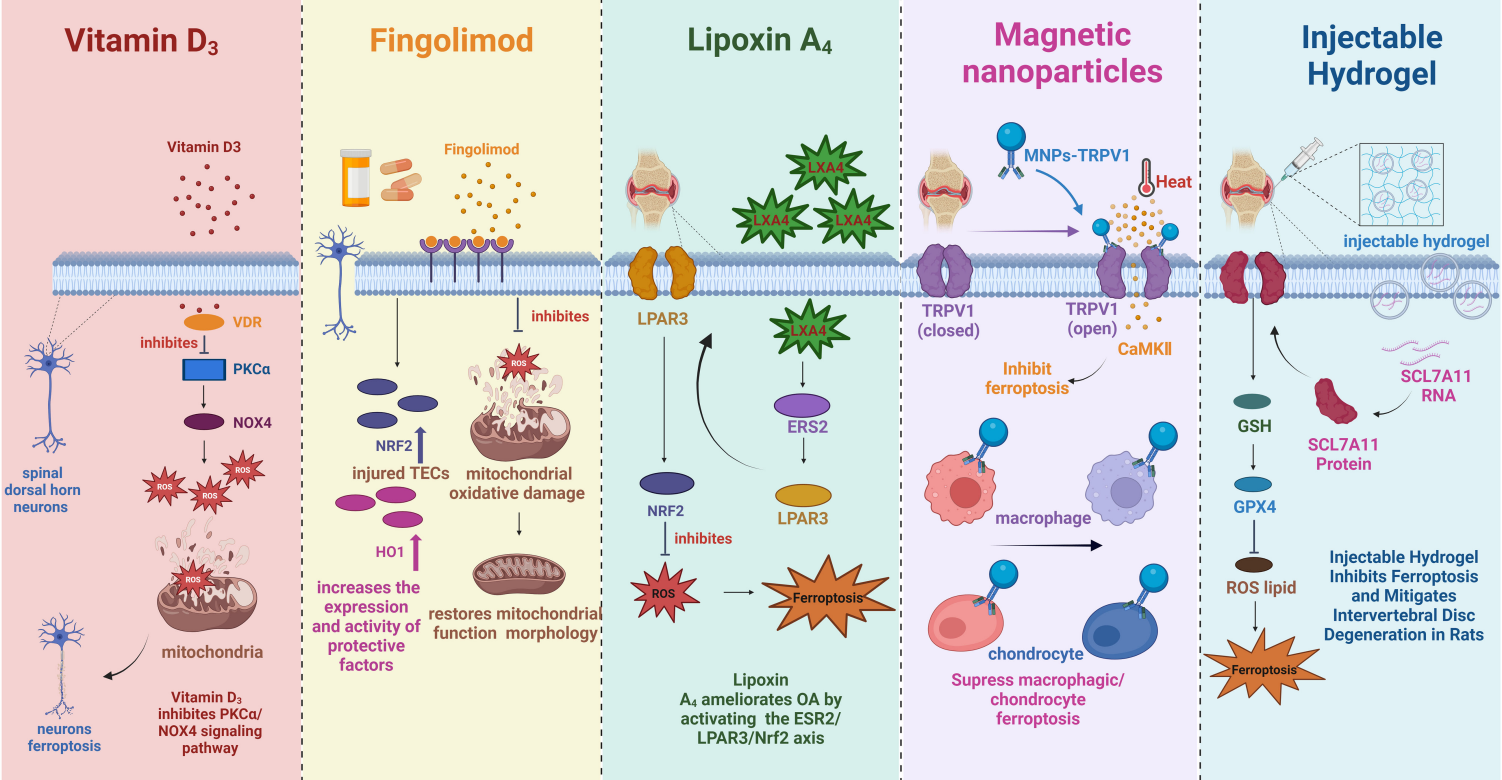
The mechanism of multiple substances to treat pain-related diseases by inhibiting ferroptosis. VDR, vitamin D receptor; PKCα, protein kinase C alpha; ROS, reactive oxygen species; NOX4, NADPH oxidase 4, Nrf2, nuclear factor E2 associated factor 2; HO1, heme oxygenase-1; LXA4, lipoxA_4_; LPAR3, lysophosphatidic acid receptor-3; ERS2, estrogen receptor beta; TRPV1, transient acceptor potential vanillic acid 1; MNPs, Magnetic nanoparticles; CaMKII, calcium/calmodulin-dependent protein kinase II; GSH, glutathione; GPX4, glutathione peroxidase 4.

In the laboratory, Vitamin D has been found to serve as an alternative treatment for NP, involving mechanisms that may be associated with ferroptosis (119, 120). VitD3 relieves NP by inhibiting mitochondria-associated ferroptosis mediated by PKCα/NOX4 signaling, preserving spinal GABaergic interneurons (**Figure 5**) (120). Used alone or in combination with existing analgesics, VitD offers an innovative treatment avenue for NP. In addition, Zhang et al. injected adenoviruses overexpressing Sirtuin 2 (Ad-Sirt2, which can regulate OS and inhibit ferroptosis) into rats to reduce iron accumulation, suppress lipid peroxidation, affect the expression of ACSL4 and GPX4, and thus alleviate chronic NP of rats (10, 121, 122). In a recent study, intraperitoneal injection of liproxstatin-1 (Lip-1, a ferroptosis inhibitor) in rats reduced iron content and lipid peroxidation in spinal cord tissue, alleviating CCI-induced mechanical and thermal nociceptive abnormalities (**Table 1**) (19). It was further found that intrathecal injection of Lip-1 efficiently reversed the rats’ mechanical nociceptive abnormalities and significantly relieved pain in a dose-dependent manner (45).

In clinical treatment, the most ordinary drug used to treat MS is glucocorticoids (GC) (123). Although rare articles directly clarify the link between GC and ferroptosis, studies have confirmed that prolonged or excessive use of GC induces mitochondrial dysfunction, resulting in increased ROS levels and decreased GSH production (124–126). In addition, other drugs used to treat MS, such as fingolimod, teriflunomide, and dimethyl fumarate, may affect the ferroptosis process to varying degrees (127). All three drugs affect OS and thus regulate the ferroptosis process. Fingolimod and teriflunomide play an antioxidant role by regulating mitochondrial OS (**Figure 5**); meanwhile, fingolimod also reduces iron deposition (128, 129). Unlike the above two drugs, dimethyl fumarate is protective in regulating ferroptosis through the Nrf2/ARE/nuclear faction-κB (NF-κB) signaling pathway (130). Similarly, inhibiting ferroptosis is also expected to improve the prognosis of SCI, focusing on targeting obstruction of lipid peroxidation and iron overload (94). Lip-1, prokineticin-2, lipoxin A4 (**Figure 5**) and NRF2 alleviate SCI by affecting lipid peroxidation processes through different mechanisms (94). For example, Lipro-1 inhibits mitochondrial lipid peroxidation, while prokineticin-2 restrains lipid peroxidation substrate synthesis, ultimately impeding lipid peroxidation (131, 132). On the other hand, iron overload is mainly treated by up-regulating ferroportin 1 and using deferoxamine (an iron chelating agent) or dynasore (a dynamin protein inhibitor) (94). Other substances that target ferroptosis for IS include natural materials (salvia miltiorrhiza, galangin, etc), drugs (edaravone, dimethyl fumarate, etc), trace elements (selenium), and synthetic substances (Fer-1) (**Table 1**) (130, 133–137). Most of them affect ferroptosis by acting on NRF2, GPX4, and ACSL4, thereby treating IS. In addition to medications, electroacupuncture (EA), a pain management method, inhibits ferroptosis by regulating OS and iron-related proteins, thereby relieving NP as well as symptoms associated with ferroptosis in the dorsal root ganglion (138, 139).

### 5.2 Bone pain-related diseases

Targeted ferroptosis for OA treatment principally focuses on reducing iron levels, and iron chelators, including deferoxamine (DFO), deferasirox (DFX), and deferiprone (DFP) have been used clinically (111, 140). The three iron chelators are used in different ways, except for DFO, administered intravenously or subcutaneously; DFX and DFP are oral formulations, and they all have been shown to chelate iron effectively and have a good safety profile (141–144). In fact, iron chelation therapy is more commonly used to treat patients with osteopenia or osteoporosis because iron chelation restrains osteoclast production and bone resorption (145, 146). Given that osteoclastogenesis of subchondral bone has been identified as a critical disruptive factor in the development and progression of OA, iron chelators hold great therapeutic promise in ferroptosis-induced OA (147). In addition, D-mannose, a ferroptosis inhibitor, can reduce the sensitivity of chondrocytes to ferroptosis and play a chondroprotective role, thereby delaying the progression of OA (**Table 1**) (148). Since lipid peroxidation-induced ferroptosis plays an important role in the cartilage degradation of OA, antioxidants, including enzymatic antioxidants (superoxide dismutases, catalase, glutathione peroxidase) and nonenzymatic antioxidants (glutathione, vitamin, coenzyme Q10), provide new ideas for OA treatment by inhibiting lipid peroxidation and ferroptosis in chondrocytes (104). In recent months, extensive experiments have emerged using natural Chinese herbs or their extracts, such as Quercetin (149), Dandouchi (150), Jianpi-Tongluo formula (151); anti-inflammatory lipid mediator, such as lipoxA_4_ (LXA_4_) (152); botulinum toxin A (BONT/A) produced by the anaerobic bacterium Clostridium botulinum (153); magnetic nanoparticles (MNP) (**Figure 5**) (154) or colloidal gold nanorods (AuNR) (155) by coupling with transient acceptor potential vanillic acid 1 (TRPV1), modulating ferroptosis to treat OA, and all have achieved excellent therapeutic effects. Similarly, the use of iron chelators (DFO) to deduce iron content, antioxidants to inhibit OS, and ferroptosis inhibitors (Fer-1) to impede ferroptosis are also feasible ways to rescue IVDD associated with iron overload (113). The specific mechanism involves DFO reversing the downregulation of GPX4 and SLC7A11, thereby blocking chondrocyte ferroptosis, while Fer-1 alleviates iron overload-induced endplate chondrocyte degeneration (113). Recently published materials for the treatment of IVDD based on ferroptosis mainly include composite biomaterials (injectable reactive oxygen species-responsive hydrogels) (**Figure 5**) (156), endogenous multifunctional and conditionally essential amino acids (glutamine) (157), novel ferroptosis inhibitors (Tinoridine) (**Table 1**) (158).

Although the number of drugs approved for treating diseases related to ferroptosis is increasing, there is still some uncertainty about these drugs’ clinical translation, mode of administration, dosage, time window of administration, and pharmacokinetics in the human body. Numerous impediments remain to be solved, and the research in this area shows a flourishing trend. At the same time, we also expect more studies to explore the relevant signaling pathways and specific mechanisms of ferroptosis’s involvement in pain to enhance the understanding of the pathogenesis of these diseases and develop new, safer, and more effective inhibitors for the treatment of ferroptosis-related pain, along with achieving clinical conversion and application, is prospective.

## 6 Conclusions and future perspectives

In recent years, the number of articles linking ferroptosis to pain-related illnesses has increased rapidly. Many crucial mechanisms have been elucidated, but a comprehensive particular opinion is far from being achieved. Exploring the mechanism of ferroptosis involved in pain and attenuating pain by targeting ferroptosis has become a prominent topic. As previously mentioned, pharmacological induction of ferroptosis is a prospective approach for treating pain-related disorders. The research and development of more medicines that target ferroptosis may significantly improve the prognosis of painful diseases. However, the method of administration, dose, time window of administration, pharmacokinetic study, and potential side effects of the drug are all urgent problems that need to be solved.

In addition, clinical treatments targeting ferroptosis continue to face multiple obstacles. On the one hand, whether ferroptosis is involved in all pain-related diseases has not been confirmed. In other words, whether a drug that targets ferroptosis would work for most pain-related diseases? On the other hand, the intricacy of the known regulatory pathways of ferroptosis makes it ineluctably function in various diseases, including cardiovascular disease, kidney disease, and cancer. The development of specific therapies to inhibit ferroptosis in painful diseases and avoid systemic adverse severe consequences are the obstacles that must be overcome to achieve clinical application. Finally, treating pain-related diseases by targeting ferroptosis is primarily performed in animal experiments, and the use of drugs targeting ferroptosis has yet to be promoted in clinical application. We expect more data from large sample populations to determine whether targeting ferroptosis can improve outcomes for painful diseases.

To our knowledge, this review is the first to summarize the molecular mechanism of ferroptosis and pain systematically, the association between ferroptosis and pain-related diseases, and the methods of targeting ferroptosis for pain-related diseases. However, our review also needs some improvement. First, we only concentrated on the association of ferroptosis with neuropathic and bone pain diseases and did not discuss other types of painful diseases connected with ferroptosis, such as cystitis/bladder pain syndrome and endometriosis. Second, the list of diseases that illustrate the association between ferroptosis and neuropathic or bone pain diseases may need to be completed. For example, the association between rheumatoid arthritis (RA), osteosarcoma, and ferroptosis is well documented, which we did not include in this review. Finally, there are few studies on ferroptosis and pain and many gaps in the specific mechanisms. The literature we can refer to is restricted. In conclusion, the vital link between pain and ferroptosis requires further investigation of the underlying mechanisms, which will hopefully boost the exploitation of novel, more refined therapeutic strategies for the handling of pain.
